# Supplementary material for: Vaccination with a Protective Ipa Protein-Containing Nanoemulsion Differentially Alters the Transcriptomic Profiles of Young and Elderly Mice following Shigella Infection
Source: Vaccines (Basel). 2024 Jun 4;12(6):618. doi: 10.3390/vaccines12060618 (PMC11209624; doi:10.3390/vaccines12060618)
Supplement: Supplementary file 1 [file vaccines-12-00618-s001.zip › vaccines-3020146-supplementary.pdf]

Supplemental Information for . . .

Vaccination with a Protective Ipa Protein-Containing Nanoemulsion Differentially Alters the Transcriptomic Profiles of Young and Elderly Mice following *Shigella* Infection

Ti Lu [1,*], Murugesan Raju [2,3], Debaki R. Howlader [1], Zackary K. Dietz [1], Sean K. Whittier [1], David J. Varisco [4], Robert K. Ernst [4], Lyndon M. Coghill [2,3], William D. Picking [1] and Wendy L. Picking [1,*]

[1] Bond Life Sciences Center and Department of Veterinary Pathobiology, University of Missouri,
Columbia, MO 65211, USA; drhb7r@missouri.edu (D.R.H.); zkdqk6@missouri.edu (Z.K.D.); sean.whittier@missouri.edu (S.K.W.); pickingw@missouri.edu (W.D.P.)
[2] Bioinformatics and Analytic Core, University of Missouri, Columbia, MO 65211, USA; rajum@health.missouri.edu (M.R.); lcoghill@missouri.edu (L.M.C.)
[3] MU Institute for Data Science and Informatics, University of Missouri, Columbia, MO 65211, USA
[4] Department of Microbial Pathogenesis, University of Maryland, Baltimore, MD 21201, USA; dvarisco@umaryland.edu (D.J.V.); rkernst@umaryland.edu (R.K.E.)
* Correspondence: tilu@missouri.edu (T.L.); wendy.picking@missouri.edu (W.L.P.)

Supplemental Table S1. Acronyms used in this paper.

T3SS	Type III secretion system
DBF	IpaD-IpaB fusion protein
dmLT	Double-mutant heat-labile enterotoxin
LTA1	A1 moiety of the active subunit of dmLT
L-DBF	LTA1 fusion with DBF
BECC	Bacterial Enzymatic Combinatorial Chemistry
ETEC	Enterotoxigenic *Escherichia coli*
IN	Intranasal
TLR-4	Toll-like receptor 4
LDAO	Lauryl-dimethylamine oxide
ME	MedImmune emulsion
MOPS	3-(N-morpholino)propanesulfonic acid
HRP	Horseradish peroxidase
OPD	O-phenylenediamine dihydrochloride
IPTG	Isopropyl β-D-1-thiogalactopyranoside
IMAC	Immobilized Metal Affinity Chromatography
MSD	Meso Scale Discovery
DEG	Differentially Expressed Gene
GO	Gene Ontology
PCA	Principal Component Analysis
EU	Endotoxin Unit
rlog	regularized log transformation
MPLA	Monophosphoryl lipid A.

Supplemental Table S2. Vaccine efficacy of an L-DBF with BECC438 or BECC470 in ME following IN administration. Young (6-8 weeks) and elderly (>18 months) mice were intranasally vaccinated with the indicated formulations (n=10). Subsequently, they were challenged with 1 X 10^6 CFU (in 30 µl) of *S. flexneri* 2a 2457T. Vaccine efficacy (VE) is presented as VE = 1 – Attack Rate Vaccinated/Attack Rate Unvaccinated (PBS control). All mice died in the control (PBS vaccinated) group.

**Vaccine Efficacy** (females, n=10 / group)	**Young** (6-8 weeks)	**Elderly** (>18 months)
BECC438/ 2 µg L-DBF/ME	100%	ND
BECC438/ 1 µg L-DBF/ME	ND*	60%
BECC470/ 2 µg L-DBF/ME	100%	ND
BECC470/ 1 µg L-DBF/ME	100%#	90%

*: ND means Not Done.

#: An additional trial to standardize the vaccine dosage for bulk mRNA-seq comparison.

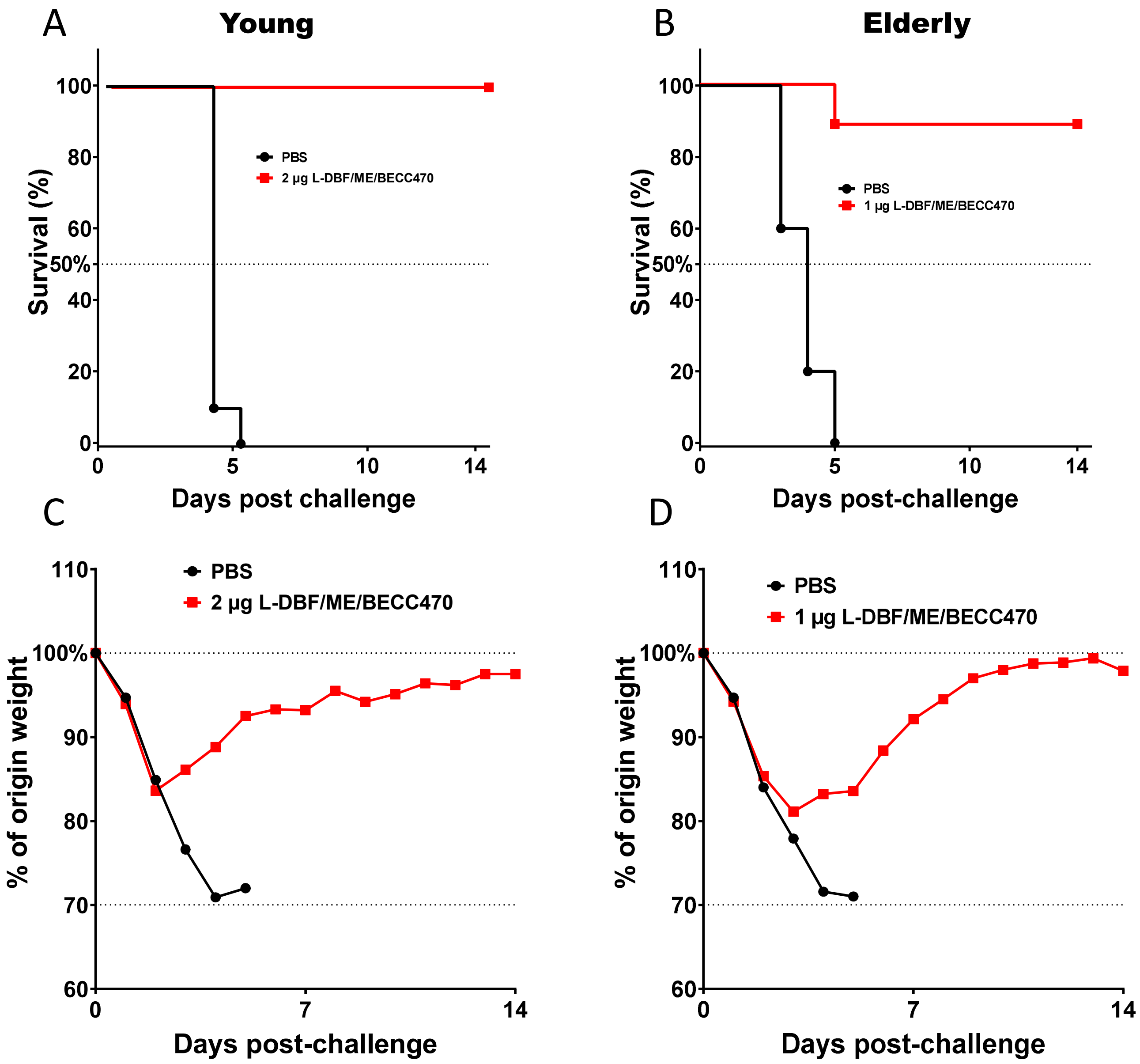

Supplemental Figure S1. Vaccination outcomes in different age groups. Young (left) and elderly (right) mice were vaccinated intranasally (IN) with PBS (Black) or L-DBF/ME/BECC470 (Red), and then challenged IN with 1 x 10^6 CFU per mouse of *S. flexneri* 2a and their weights monitored every 24 h for 14 days. **Top panels** present survival data in young (A) or elderly (B) mice following *S. flexneri* 2a challenge with 1 x 10^6 CFU/mouse. **Bottom panels** show the weight loss of young (C) or elderly (D) mice following *Shigella* challenge.

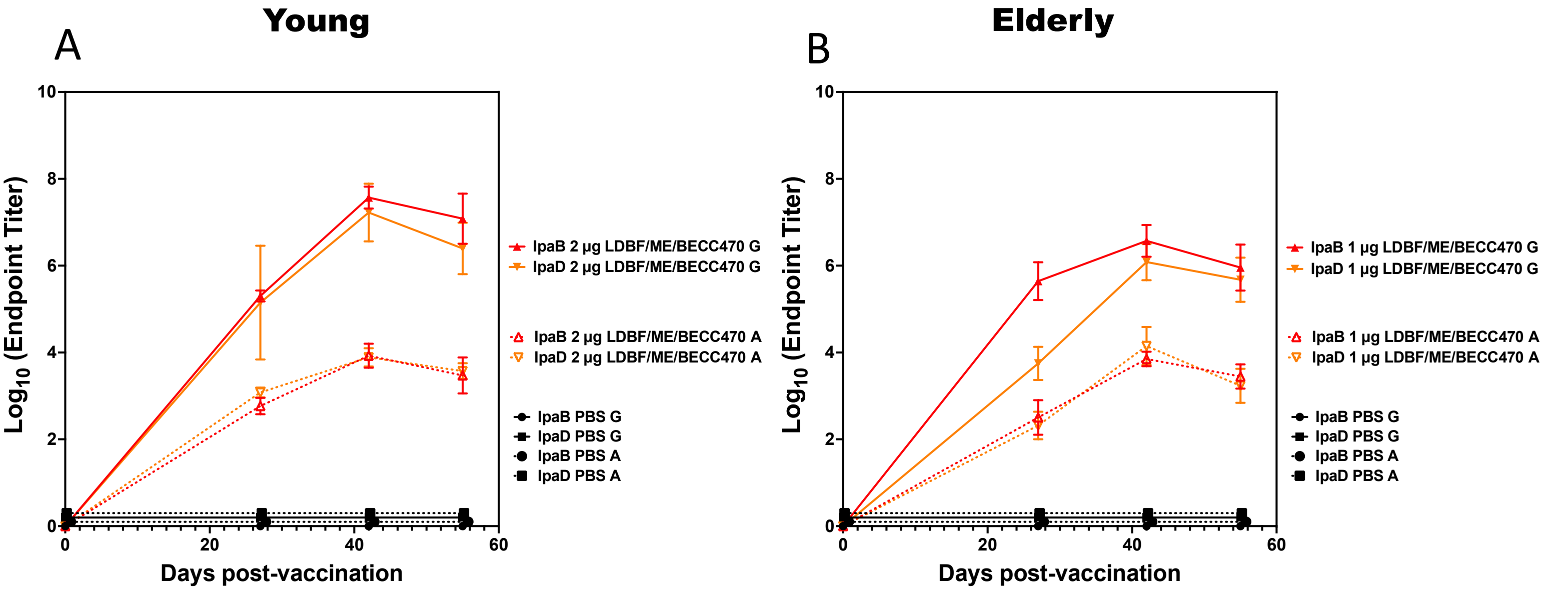

Supplemental Figure S2. Antigen-specific IgG and IgA responses. Young **(A)** and elderly **(B)** mice were vaccinated intranasally (IN) with PBS or L-DBF in BECC470/ME on days 0, 14, and 28. Blood and fecal samples were collected and serum titers for serum IgG (filled symbols with solid lines; G) and fecal IgA (open symbols with dashed lines; A) specific for IpaB (Red) or IpaD (Yellow) were measured by ELISA. The individual titers are represented as EU ml-1. Each point represents the mean and error bars represent SD of each group (n=10/group).

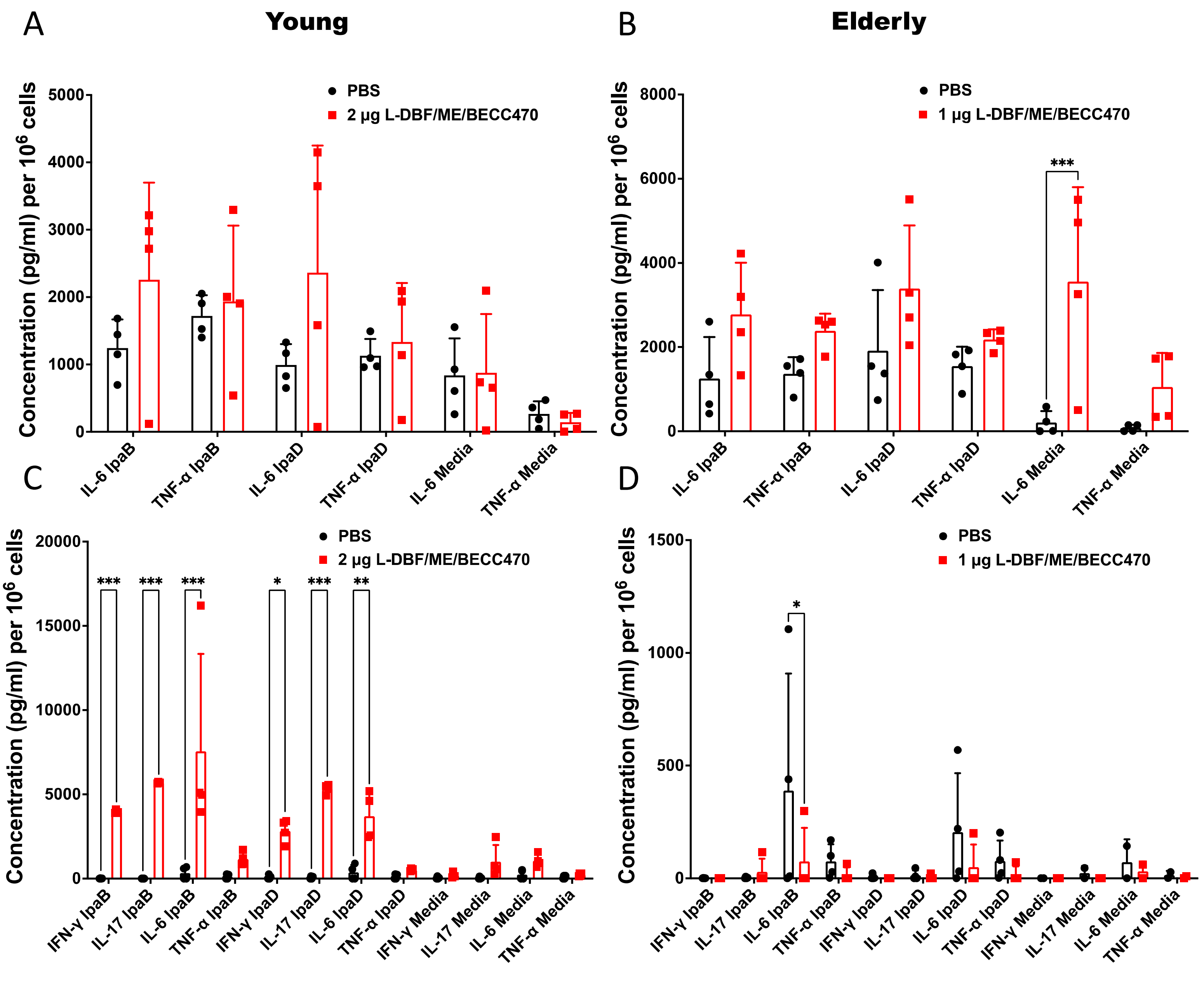

Supplemental Figure S3. Cytokine secretion from lung cells (**A & B**) or splenocytes (**C & D**) prepared from young (Left) and elderly (Right) mice vaccinated intranasally (IN) with PBS (Black) or L-DBF in BECC470/ME (Red). All samples were collected on day 3 before the challenge. Single-cell lung suspensions were incubated with 10 µg/ml IpaB and IpaD. Cytokine levels were determined by Meso Scale Discovery analysis as per the manufacturer's specifications and are presented here as pg/ml/10^6 cells. Secretion of different cytokines was noted as a response of either IpaB or IpaD stimulation. Data were plotted as actual values from individuals ± SD (n = 4) in each group. Significance was calculated by comparing groups that were unvaccinated (PBS) and mice vaccinated with antigens using a Welch t-test. *p<0.05; **p<0.01; ***p< 0.001.

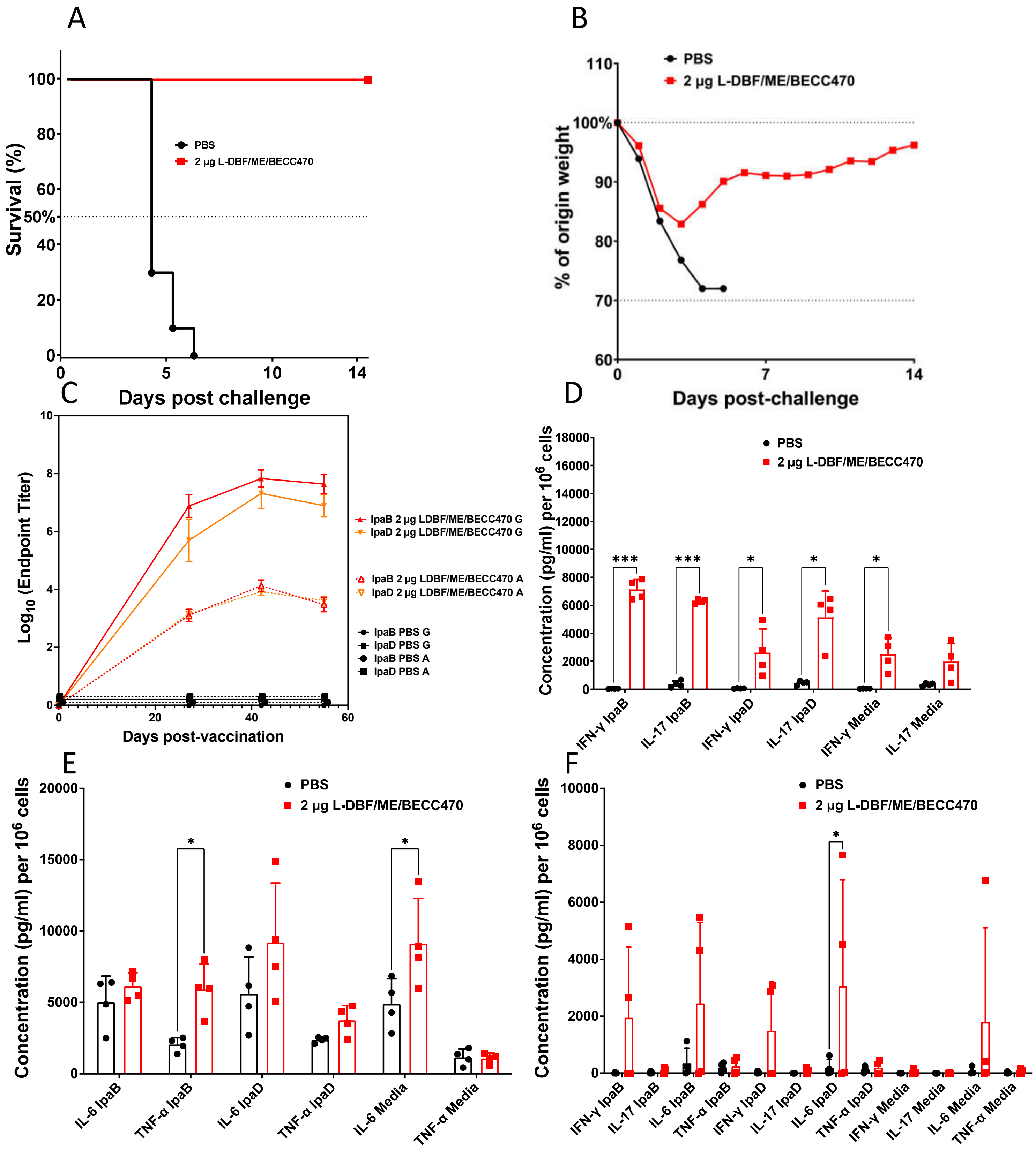

A
Survival (%)
100
80
60
50%
40
20
0
0
5
10
14
Days post challenge
PBS
2 µg L-DBF/ME/BECC470
B
110
100%
90
80
70
60
% of origin weight
0
7
14
Days post-challenge
PBS
2 µg L-DBF/ME/BECC470
C
Log10 (Endpoint Titer)
10
8
6
4
2
0
0
20
40
60
Days post-vaccination
IpaB 2 µg LDBF/ME/BECC470 G
IpaD 2 µg LDBF/ME/BECC470 G
IpaB 2 µg LDBF/ME/BECC470 A
IpaD 2 µg LDBF/ME/BECC470 A
IpaB PBS G
IpaD PBS G
IpaB PBS A
IpaD PBS A
D
Concentration (pg/ml) per 10^6 cells
18000
16000
14000
12000
10000
8000
6000
4000
2000
0
PBS
2 µg L-DBF/ME/BECC470
***
***
*
*
*
IFN-γ IpaB
IL-17 IpaB
IFN-γ IpaD
IL-17 IpaD
IFN-γ Media
IL-17 Media
E
Concentration (pg/ml) per 10^6 cells
20000
15000
10000
5000
0
PBS
2 µg L-DBF/ME/BECC470
*
*
IL-6 IpaB
TNF-α IpaB
IL-6 IpaD
TNF-α IpaD
IL-6 Media
TNF-α Media
F
Concentration (pg/ml) per 10^6 cells
10000
8000
6000
4000
2000
0
PBS
2 µg L-DBF/ME/BECC470
*
IFN-γ IpaB
IL-17 IpaB
IL-6 IpaB
TNF-α IpaB
IFN-γ IpaD
IL-17 IpaD
IL-6 IpaD
TNF-α IpaD
IFN-γ Media
IL-17 Media
IL-6 Media
TNF-α Media

Supplemental Figure S4. Vaccination outcomes in mature age groups. Mature mice (n=10 / group) were vaccinated intranasally (IN) with PBS (Black) or L-DBF/ME/BECC470 (Red). **Top panels** present survival data (A) and weight loss curve (B) following *S. flexneri 2a* challenge with 1 x 10^6 CFU/mouse. **Panel C** shows antigen-specific serum IgG (filled symbols with solid lines; G) and fecal IgA (open symbols with dashed lines; A) responses from mature age groups. Titers specific for IpaB (Red) or IpaD (Yellow) were measured by ELISA. The individual titers are represented as EU ml-1. Each point represents the mean and error bars represent SD of each group. Cytokine levels in suspensions of lung cells **(D & E)** or splenocytes **(F)** collected on day 3 before the challenge for mature mice were displayed. Cell suspensions were incubated with 10 µg/ml IpaB and IpaD. Cytokine levels were determined by Meso Scale Discovery analysis as per the manufacturer's specifications and are presented here as pg/ml/10^6 cells. Secretion to different cytokines was noted as a response of either IpaB or IpaD stimulation. Data were plotted as actual values from individuals ± SD (n = 4) in each group. Significance was calculated by comparing groups that were unvaccinated (PBS) and mice vaccinated with antigens using a Welch t-test. *p<0.05; **p<0.01; ***p< 0.001.

A

Young

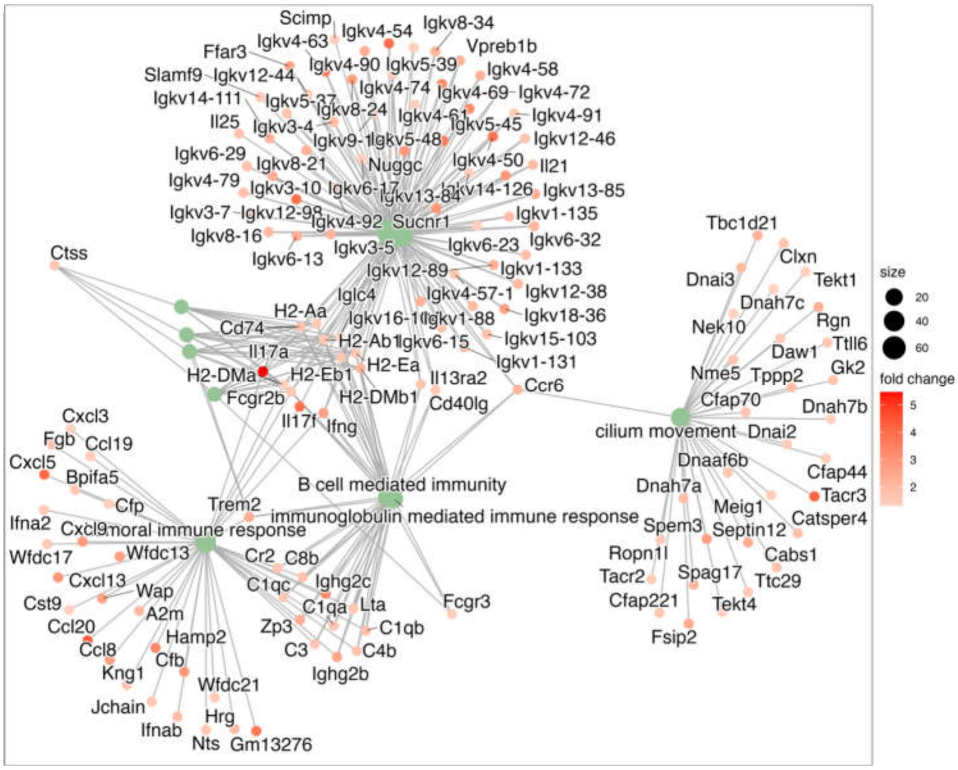
Scimp
Igkv8-34
Igkv4-54
Igkv4-63
Ffar3
Vpreb1b
Igkv4-90
Igkv5-39
Igkv4-58
Slamf9
Igkv12-44
Igkv4-74
Igkv4-69
Igkv4-72
Igkv14-111
Igkv8-24
Igkv4-61
Igkv4-91
Il25
Igkv3-4
Igkv5-45
Igkv5-48
Igkv12-46
Igkv6-29
Igkv8-21
Nuggc
Igkv4-50
Il21
Igkv4-79
Igkv3-10
Igkv13-85
Igkv3-7
Igkv12-98
Igkv4-92
Sucnr1
Igkv1-135
Igkv8-16
Igkv6-23
Igkv6-32
Igkv3-5
Ctss
Igkv6-13
Igkv12-89
Igkv1-133
Tbc1d21
Clxn
Tekt1
Dnai3
Dnah7c
Igkv4-57-1
Igkv12-38
Iglc4
H2-Aa
Igkv18-36
Nek10
Rgn
Cd74
Igkv1-88
Igkv6-15
Igkv15-103
Daw1
Ttll6
Il17a
H2-Ab1
Gk2
H2-Ea
Igkv1-131
Nme5
Tppp2
H2-DMa
H2-Eb1
Il13ra2
Ccr6
Cfap70
Dnah7b
Fcgr2b
H2-DMb1
Cd40lg
Cxcl3
Il17f
Ifng
cilium movement
Dnai2
Fgb
Ccl19
Dnaaf6b
Cxcl5
Cfap44
Bpifa5
B cell mediated immunity
Dnah7a
Tacr3
Cfp
Trem2
Meig1
Ifna2
immunoglobulin mediated immune response
Catsper4
Spem3
Septin12
Wfdc17
Wfdc13
Cr2
C8b
Ropn1l
Cabs1
Cxcl13
Ighg2c
Tacr2
Spag17
Ttc29
Cst9
C1qc
Wap
C1qa
Lta
Fcgr3
Cfap221
Tekt4
A2m
Ccl20
Zp3
C1qb
Hamp2
Fsip2
Ccl8
Cfb
C3
C4b
Kng1
Ighg2b
Wfdc21
Jchain
Hrg
Ifnab
Nts
Gm13276
size
20
40
60
fold change
5
4
3
2

B

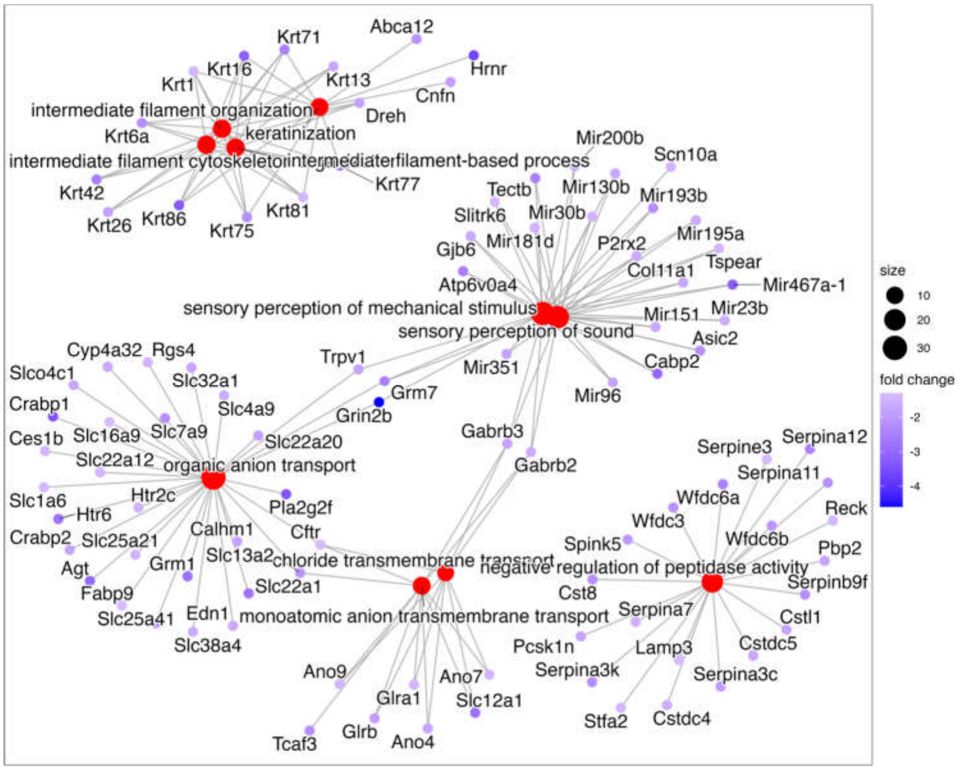
Abca12
Krt71
Hrnr
Krt16
Krt1
Krt13
Cnfn
intermediate filament organization
Dreh
Krt6a
keratinization
Mir200b
intermediate filament-based process
Scn10a
Krt77
Tectb
Mir130b
Mir193b
Krt42
Krt86
Krt81
Slitrk6
Mir30b
Krt26
Krt75
Mir195a
Gjb6
Mir181d
P2rx2
Col11a1
Tspear
Atp6v0a4
Mir467a-1
sensory perception of mechanical stimulus
Mir23b
Mir151
sensory perception of sound
Asic2
Cyp4a32
Rgs4
Trpv1
Cabp2
Slco4c1
Slc32a1
Mir351
Grm7
Mir96
Crabp1
Slc4a9
Grin2b
Ces1b
Slc16a9
Slc7a9
Slc22a20
Gabrb3
Serpine3
Serpina12
Slc22a12
organic anion transport
Gabrb2
Serpina11
Slc1a6
Htr2c
Wfdc6a
Htr6
Pla2g2f
Wfdc3
Reck
Calhm1
Cftr
Spink5
Wfdc6b
Crabp2
Slc25a21
Pbp2
Agt
Grm1
Slc13a2
chloride transmembrane transport
negative regulation of peptidase activity
Serpinb9f
Fabp9
Slc22a1
Cst8
Slc25a41
Edn1
monoatomic anion transmembrane transport
Serpina7
Cstl1
Slc38a4
Pcsk1n
Lamp3
Cstdc5
Ano9
Ano7
Serpina3k
Serpina3c
Glra1
Slc12a1
Stfa2
Cstdc4
Tcaf3
Glrb
Ano4
size
10
20
30
fold change
-2
-3
-4

Supplemental Figure S5. GO Enrichment Analysis of differential gene expression clusters in response to L-DBF/ME/ BECC470 in young mice. Cluster images representing upregulated genes (log2FoldChange>1.3; Panel A) and downregulated genes (log2FoldChange < -1.3; Panel B). Each dot corresponds to a gene, and each cluster spot represents a related pathway, with the size to show how many genes clustered. The color intensity indicates the expression level, with heavier colors indicating higher (A) or lower (B) expression. Genes are clustered based on similar expression patterns across conditions.

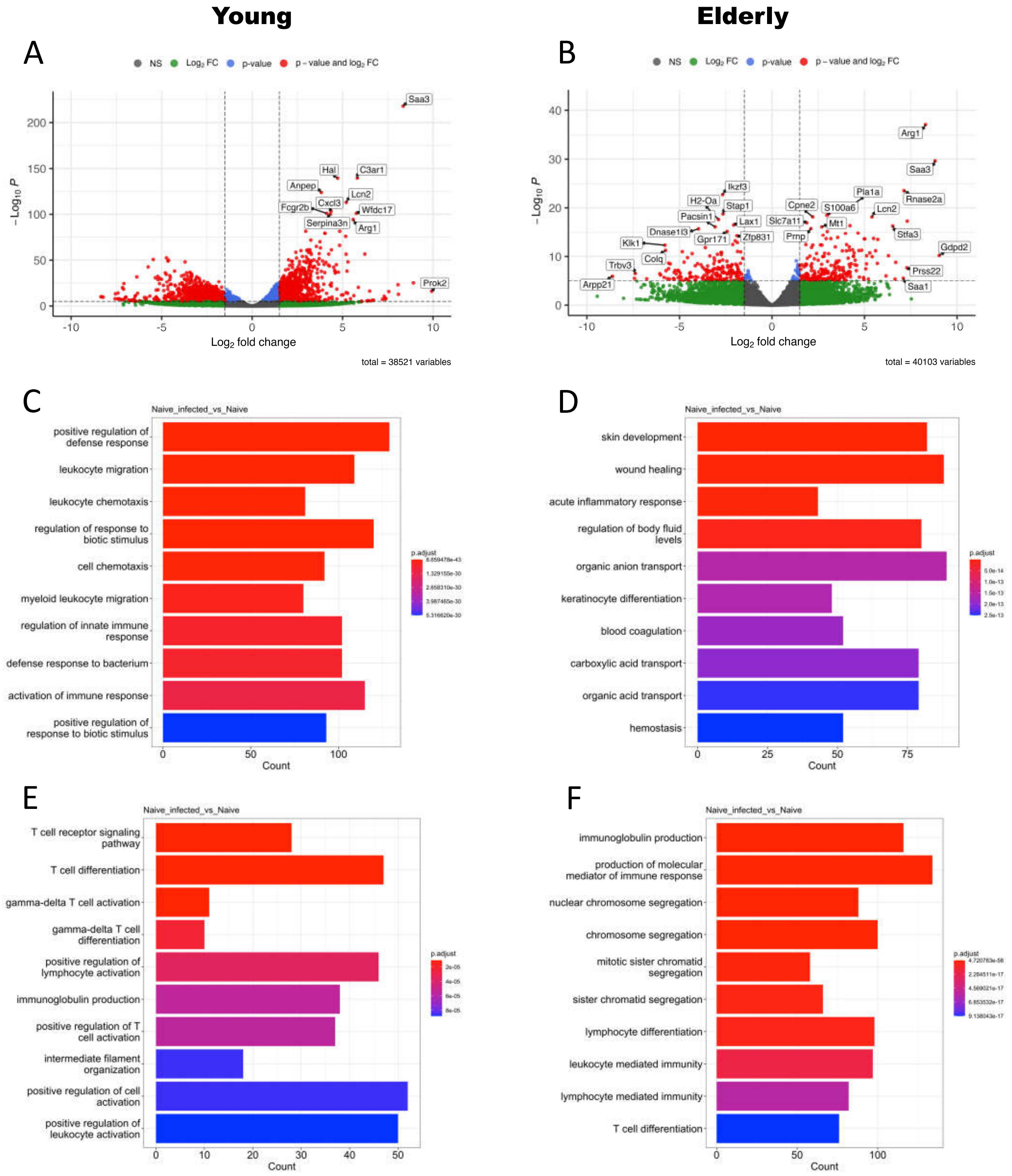

Young
Elderly
A
B
NS
Log2 FC
p-value
p – value and log2 FC
– Log10 P
Log2 fold change
Saa3
Hal
C3ar1
Anpep
Lcn2
Cxcl3
Fcgr2b
Wfdc17
Serpina3n
Arg1
Prok2
total = 38521 variables
Arg1
Saa3
Ikzf3
Pla1a
Rnase2a
H2-Oa
Stap1
Cpne2
S100a6
Lcn2
Pacsin1
Lax1
Slc7a11
Mt1
Dnase1l3
Gpr171
Zfp831
Prnp
Stfa3
Klk1
Gdpd2
Colq
Trbv3
Prss22
Arpp21
Saa1
total = 40103 variables
C
D
Naive_infected_vs_Naive
positive regulation of defense response
leukocyte migration
leukocyte chemotaxis
regulation of response to biotic stimulus
cell chemotaxis
myeloid leukocyte migration
regulation of innate immune response
defense response to bacterium
activation of immune response
positive regulation of response to biotic stimulus
Count
p.adjust
skin development
wound healing
acute inflammatory response
regulation of body fluid levels
organic anion transport
keratinocyte differentiation
blood coagulation
carboxylic acid transport
organic acid transport
hemostasis
E
F
T cell receptor signaling pathway
T cell differentiation
gamma-delta T cell activation
gamma-delta T cell differentiation
positive regulation of lymphocyte activation
immunoglobulin production
positive regulation of T cell activation
intermediate filament organization
positive regulation of cell activation
positive regulation of leukocyte activation
immunoglobulin production
production of molecular mediator of immune response
nuclear chromosome segregation
chromosome segregation
mitotic sister chromatid segregation
sister chromatid segregation
lymphocyte differentiation
leukocyte mediated immunity
lymphocyte mediated immunity
T cell differentiation

Supplemental Figure S6. Integrated analysis of differentially expressed genes and pathway enrichment in naïve mice at young (left) or elderly (right) age in response to *Shigella* infection (NI vs. N). **Top panels**: Volcano images illustrating the distribution of gene expression changes in naïve young (A) or elderly (B) mice in response to *S. flexneri* 2a infection. Each data point represents a gene, with colors indicating statistical significance and fold change. Grey spots denote non-significant changes (NS), green spots signify $|log2FoldChange| > 2$ with $p > 0.05$, blue spots represent $p < 0.05$ and $-2 < log2FoldChange < 2$, and red spots highlight significantly changed genes with $p < 0.05$ and $|log2FoldChange| > 2$. **Middle panels**: Top 10 upregulated pathways based on Gene Ontology (GO) enrichment analysis in naïve young (C) or elderly (D) mice in response to *S. flexneri* 2a infection compared to their N groups. These pathways provide insights into the biological processes associated with the upregulated genes. **Bottom panels**: Top 10 downregulated pathways based on GO enrichment analysis in naïve young (E) or elderly (F) mice in response to *S. flexneri* 2a infection compared to their N groups. These pathways reveal the biological processes affected by the downregulated genes.

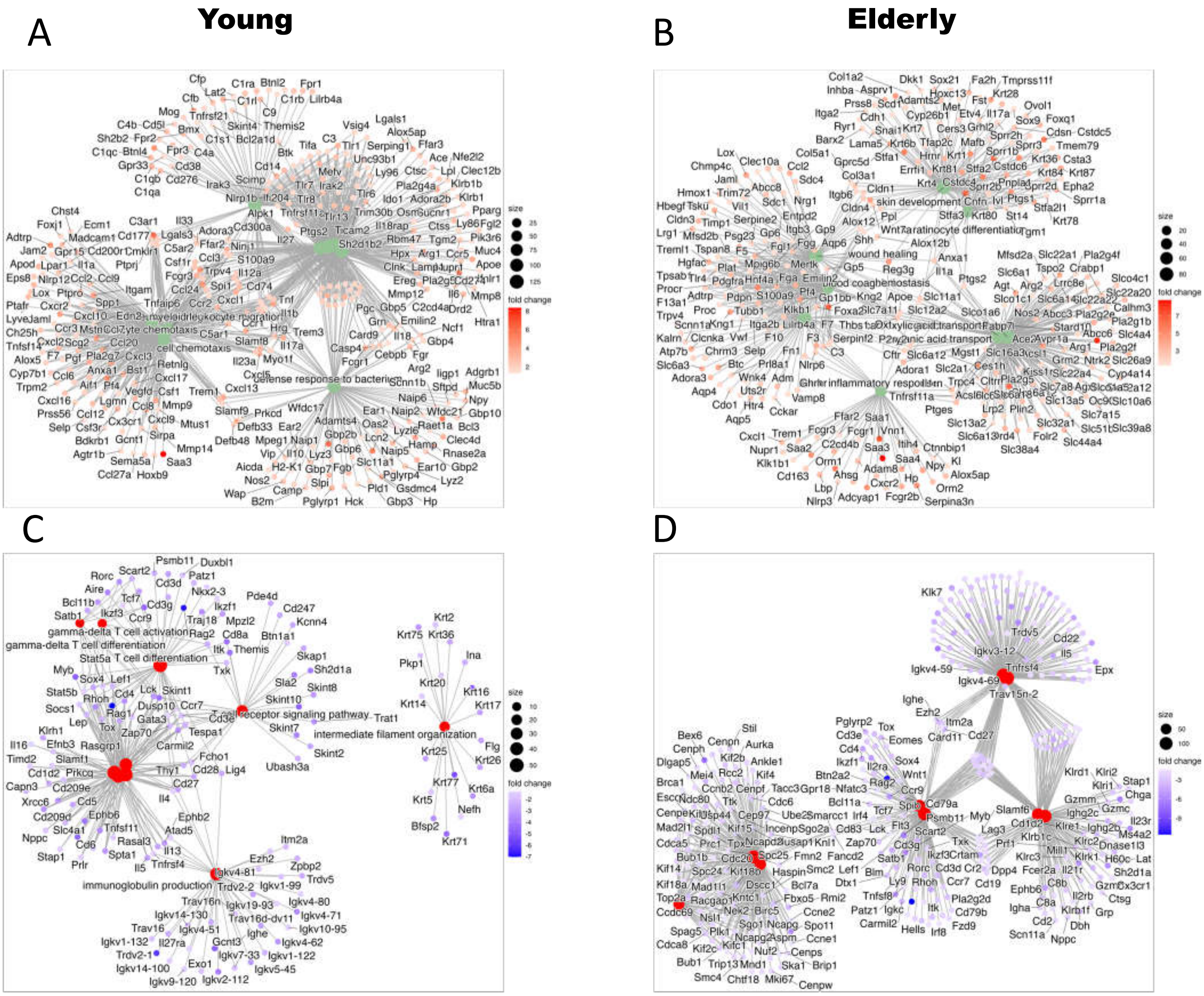

Supplemental Figure S7. Integrated analysis of differentially expressed genes and pathway enrichment in naïve mice at young (left) or elderly (right) age in response to *Shigella* infection (NI vs. N). **Top panels** show the cluster images highlighting upregulated genes (log2FoldChange > 1.3) identified through differential expression analysis in young (A) or elderly (B) NI mice compared to their N groups. The color intensity represents gene expression levels, with each dot corresponding to a gene and each cluster spot to a related pathway. **Bottom panels** display the cluster images illustrating downregulated genes (log2FoldChange < -1.3) identified through differential expression analysis in young (C) or elderly (D) NI mice compared to their N groups. The color scale indicates expression levels, with genes clustered based on shared expression patterns.

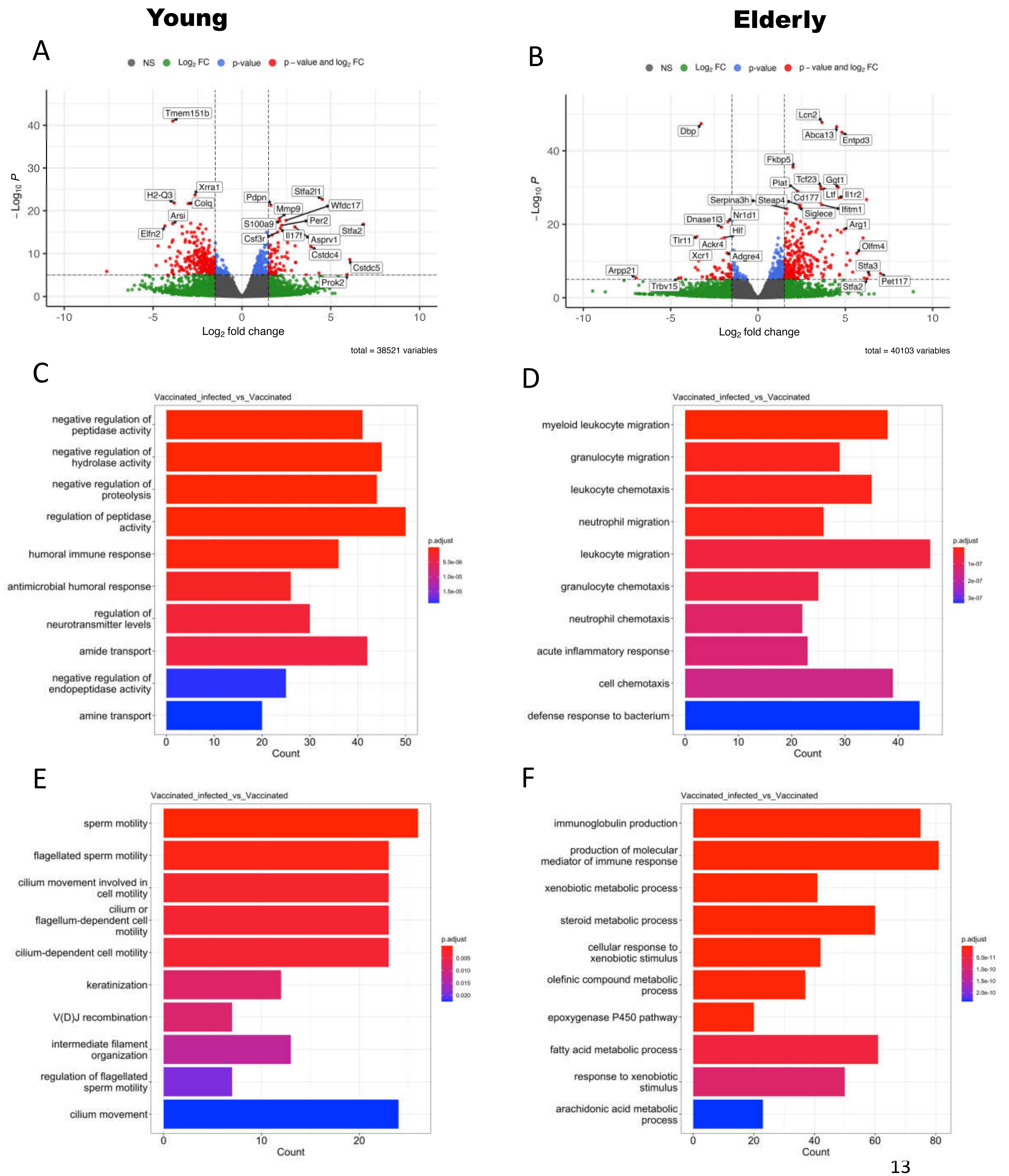
Young
Elderly
A
NS
Log2 FC
p-value
p – value and log2 FC
Tmem151b
H2-Q3
Xrra1
Colq
Arsi
Elfn2
Pdpn
Stfa2l1
Wfdc17
Mmp9
S100a9
Per2
Csf3r
Il17f
Stfa2
Asprv1
Cstdc4
Cstdc5
Prok2
– Log10 P
Log2 fold change
total = 38521 variables
B
Dbp
Lcn2
Abca13
Entpd3
Fkbp5
Plat
Tcf23
Ggt1
Serpina3h
Steap4
Cd177
Ltf
Il1r2
Siglece
Ifitm1
Dnase1l3
Nr1d1
Hlf
Arg1
Tlr11
Ackr4
Xcr1
Adgre4
Olfm4
Arpp21
Trbv15
Stfa3
Pet117
Stfa2
total = 40103 variables
C
Vaccinated_infected_vs_Vaccinated
negative regulation of peptidase activity
negative regulation of hydrolase activity
negative regulation of proteolysis
regulation of peptidase activity
humoral immune response
antimicrobial humoral response
regulation of neurotransmitter levels
amide transport
negative regulation of endopeptidase activity
amine transport
Count
p.adjust
5.0e-06
1.0e-05
1.5e-05
D
Vaccinated_infected_vs_Vaccinated
myeloid leukocyte migration
granulocyte migration
leukocyte chemotaxis
neutrophil migration
leukocyte migration
granulocyte chemotaxis
neutrophil chemotaxis
acute inflammatory response
cell chemotaxis
defense response to bacterium
Count
p.adjust
1e-07
2e-07
3e-07
E
Vaccinated_infected_vs_Vaccinated
sperm motility
flagellated sperm motility
cilium movement involved in cell motility
cilium or flagellum-dependent cell motility
cilium-dependent cell motility
keratinization
V(D)J recombination
intermediate filament organization
regulation of flagellated sperm motility
cilium movement
Count
p.adjust
0.005
0.010
0.015
0.020
F
Vaccinated_infected_vs_Vaccinated
immunoglobulin production
production of molecular mediator of immune response
xenobiotic metabolic process
steroid metabolic process
cellular response to xenobiotic stimulus
olefinic compound metabolic process
epoxygenase P450 pathway
fatty acid metabolic process
response to xenobiotic stimulus
arachidonic acid metabolic process
Count
p.adjust
5.0e-11
1.0e-10
1.5e-10
2.0e-10

Supplemental Figure S8. Integrated analysis of differentially expressed genes and pathway enrichment in mice vaccinated with L-DBF/ME/ BECC470 at young (left) or elderly (right) age in response to *Shigella* infection (VI vs. V). **Top panels**: Volcano images illustrating the distribution of gene expression changes in vaccinated young (A) or elderly (B) mice in response to *S. flexneri* 2a infection. Each data point represents a gene, with colors indicating statistical significance and fold change. Grey spots denote non-significant changes (NS), green spots signify |log2FoldChange| > 2 with $p > 0.05$, blue spots represent $p < 0.05$ and -2 < log2FoldChange < 2, and red spots highlight significantly changed genes with $p < 0.05$ and |log2FoldChange| > 2. **Middle panels**: Top 10 upregulated pathways based on Gene Ontology (GO) enrichment analysis in vaccinated young (C) or elderly (D) mice in response to *S. flexneri* 2a infection compared to their V groups. These pathways provide insights into the biological processes associated with the upregulated genes. **Bottom panels**: Top 10 downregulated pathways based on GO enrichment analysis in vaccinated young (E) or elderly (F) mice in response to *S. flexneri* 2a infection compared to their V groups. These pathways reveal the biological processes affected by the downregulated genes.

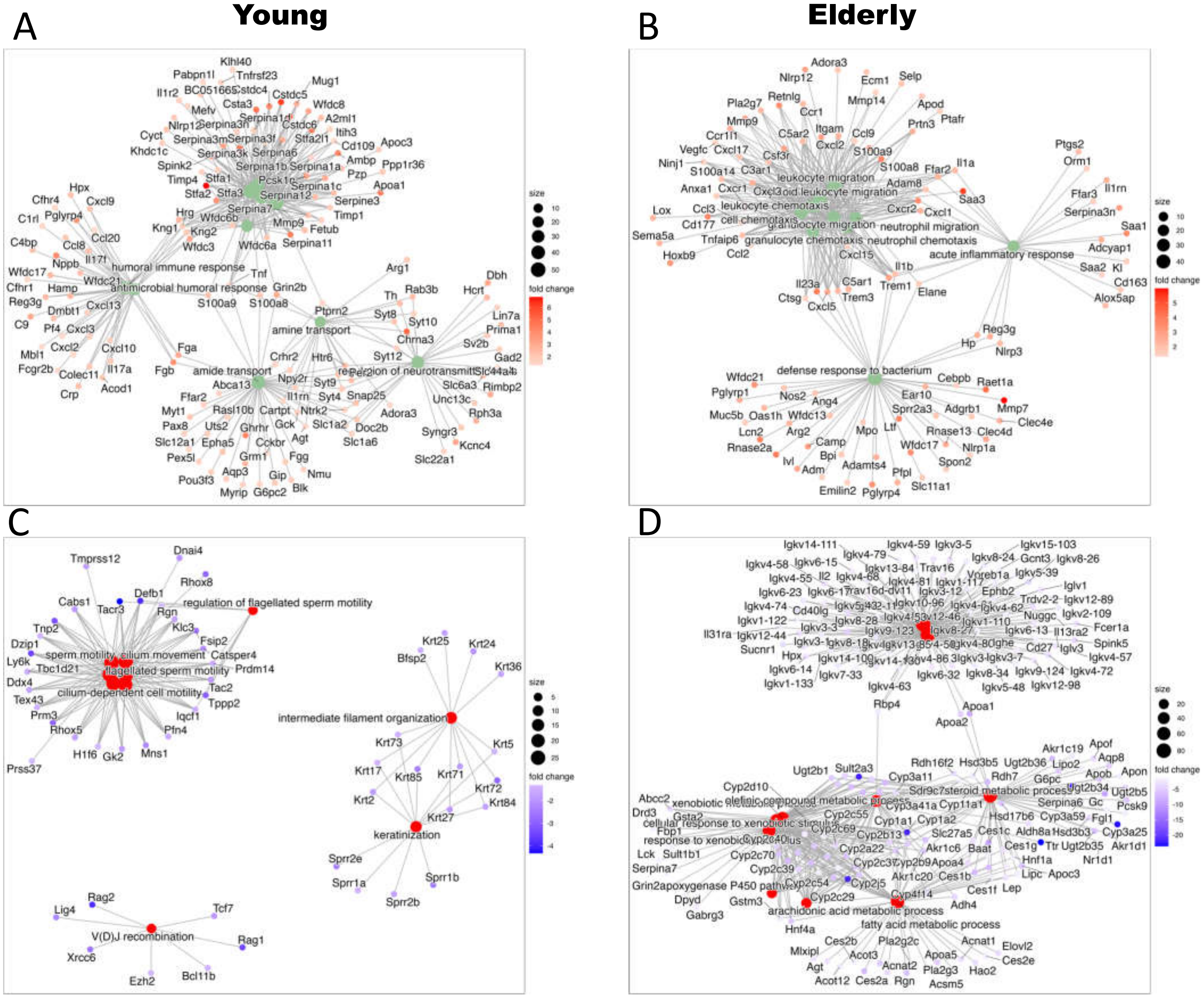

Supplemental Figure S9. Integrated analysis of differentially expressed genes and pathway enrichment in mice vaccinated with L-DBF/ME/ BECC470 at young (left) or elderly (right) age in response to *Shigella* infection (VI vs. V). **Top panels**: Cluster images presenting upregulated genes (log2FoldChange > 1.3) identified through differential expression analysis in vaccinated young (A) or elderly (B) mice in response to *S. flexneri* 2a infection. Each dot corresponds to a gene, and clusters indicate related pathways. Color intensity represents gene expression levels. **Bottom panels**: Cluster images illustrating downregulated genes (log2FoldChange < -1.3) identified through differential expression analysis in vaccinated young (C) or elderly (D) mice in response to *S. flexneri* 2a infection. Gene clusters are based on shared expression patterns, and color intensity indicates expression levels.

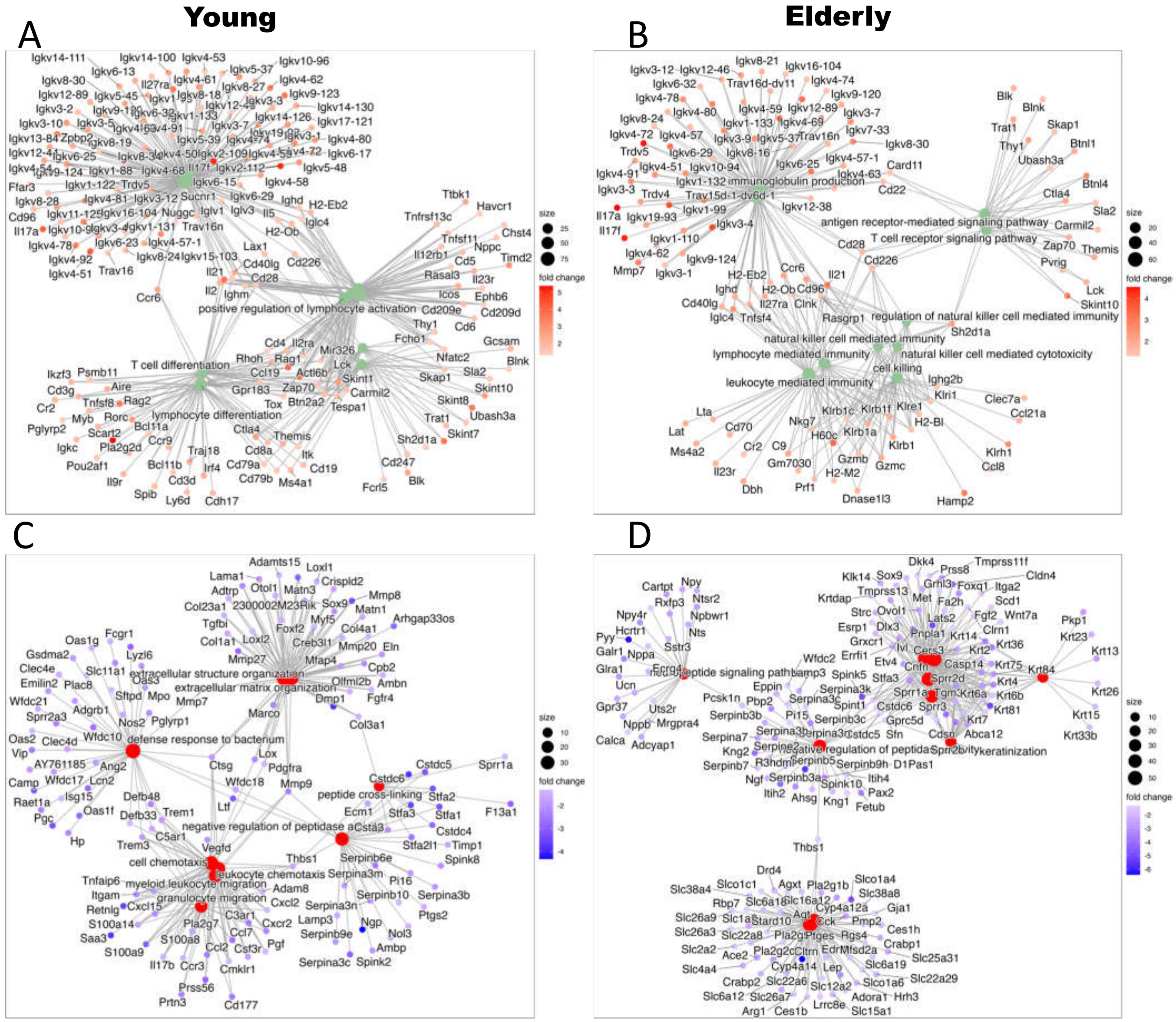

Supplemental Figure S10. Integrated analysis of differentially expressed genes and pathway enrichment in mice vaccinated with L-DBF/ME/ BECC470 at young (left) or elderly (right) age in response to *Shigella* infection (VI vs. NI). **Top panels** show the cluster images highlighting upregulated genes (log2FoldChange > 1.3) identified through differential expression analysis in young (A) or elderly (B) VI mice compared to their NI groups. The color intensity represents gene expression levels, with each dot corresponding to a gene and each cluster spot to a related pathway. **Bottom panels** display the cluster images illustrating downregulated genes (log2FoldChange < -1.3) identified through differential expression analysis in young (C) or elderly (D) VI mice compared to their NI groups. The color scale indicates expression levels, with genes clustered based on shared expression patterns.

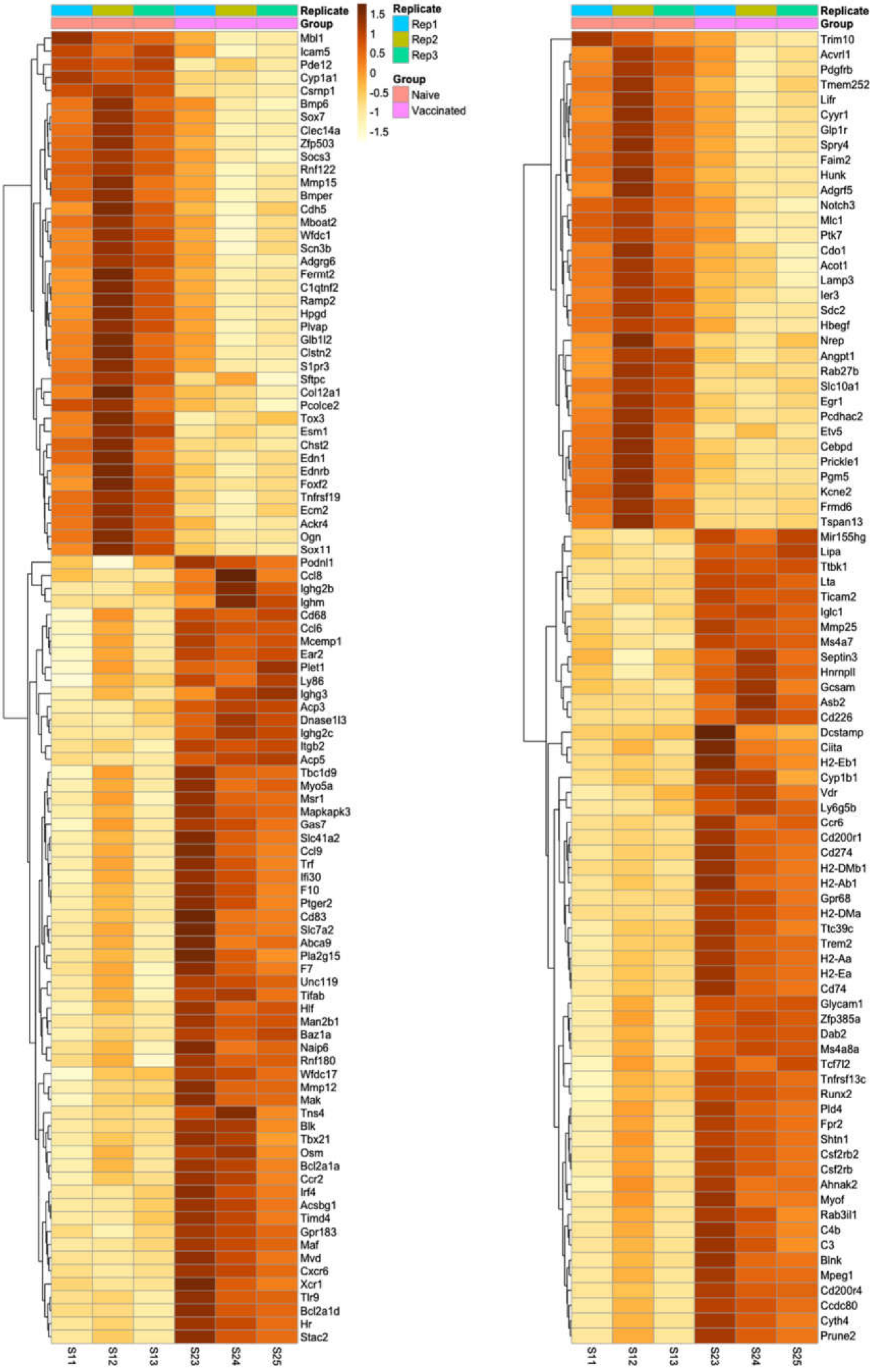
Replicate
Group
Mbl1
Icam5
Pde12
Cyp1a1
Csrnp1
Bmp6
Sox7
Clec14a
Zfp503
Socs3
Rnf122
Mmp15
Bmper
Cdh5
Mboat2
Wfdc1
Scn3b
Adgrg6
Fermt2
C1qtnf2
Ramp2
Hpgd
Plvap
Glb1l2
Clstn2
S1pr3
Sftpc
Col12a1
Pcolce2
Tox3
Esm1
Chst2
Edn1
Ednrb
Foxf2
Tnfrsf19
Ecm2
Ackr4
Ogn
Sox11
Podnl1
Ccl8
Ighg2b
Ighm
Cd68
Ccl6
Mcemp1
Ear2
Plet1
Ly86
Ighg3
Acp3
Dnase1l3
Ighg2c
Itgb2
Acp5
Tbc1d9
Myo5a
Msr1
Mapkapk3
Gas7
Slc41a2
Ccl9
Trf
Ifi30
F10
Ptger2
Cd83
Slc7a2
Abca9
Pla2g15
F7
Unc119
Tifab
Hlf
Man2b1
Baz1a
Naip6
Rnf180
Wfdc17
Mmp12
Mak
Tns4
Blk
Tbx21
Osm
Bcl2a1a
Ccr2
Irf4
Acsbg1
Timd4
Gpr183
Maf
Mvd
Cxcr6
Xcr1
Tlr9
Bcl2a1d
Hr
Stac2
S11
S12
S13
S23
S24
S25
1.5
1
0.5
0
-0.5
-1
-1.5
Replicate
Rep1
Rep2
Rep3
Group
Naive
Vaccinated
Replicate
Group
Trim10
Acvrl1
Pdgfrb
Tmem252
Lifr
Cyyr1
Glp1r
Spry4
Faim2
Hunk
Adgrf5
Notch3
Mlc1
Ptk7
Cdo1
Acot1
Lamp3
Ier3
Sdc2
Hbegf
Nrep
Angpt1
Rab27b
Slc10a1
Egr1
Pcdhac2
Etv5
Cebpd
Prickle1
Pgm5
Kcne2
Frmd6
Tspan13
Mir155hg
Lipa
Ttbk1
Lta
Ticam2
Iglc1
Mmp25
Ms4a7
Septin3
Hnrnpll
Gcsam
Asb2
Cd226
Dcstamp
Ciita
H2-Eb1
Cyp1b1
Vdr
Ly6g5b
Ccr6
Cd200r1
Cd274
H2-DMb1
H2-Ab1
Gpr68
H2-DMa
Ttc39c
Trem2
H2-Aa
H2-Ea
Cd74
Glycam1
Zfp385a
Dab2
Ms4a8a
Tcf7l2
Tnfrsf13c
Runx2
Pld4
Fpr2
Shtn1
Csf2rb2
Csf2rb
Ahnak2
Myof
Rab3il1
C4b
C3
Blnk
Mpeg1
Cd200r4
Ccdc80
Cyth4
Prune2
S11
S12
S13
S23
S24
S25

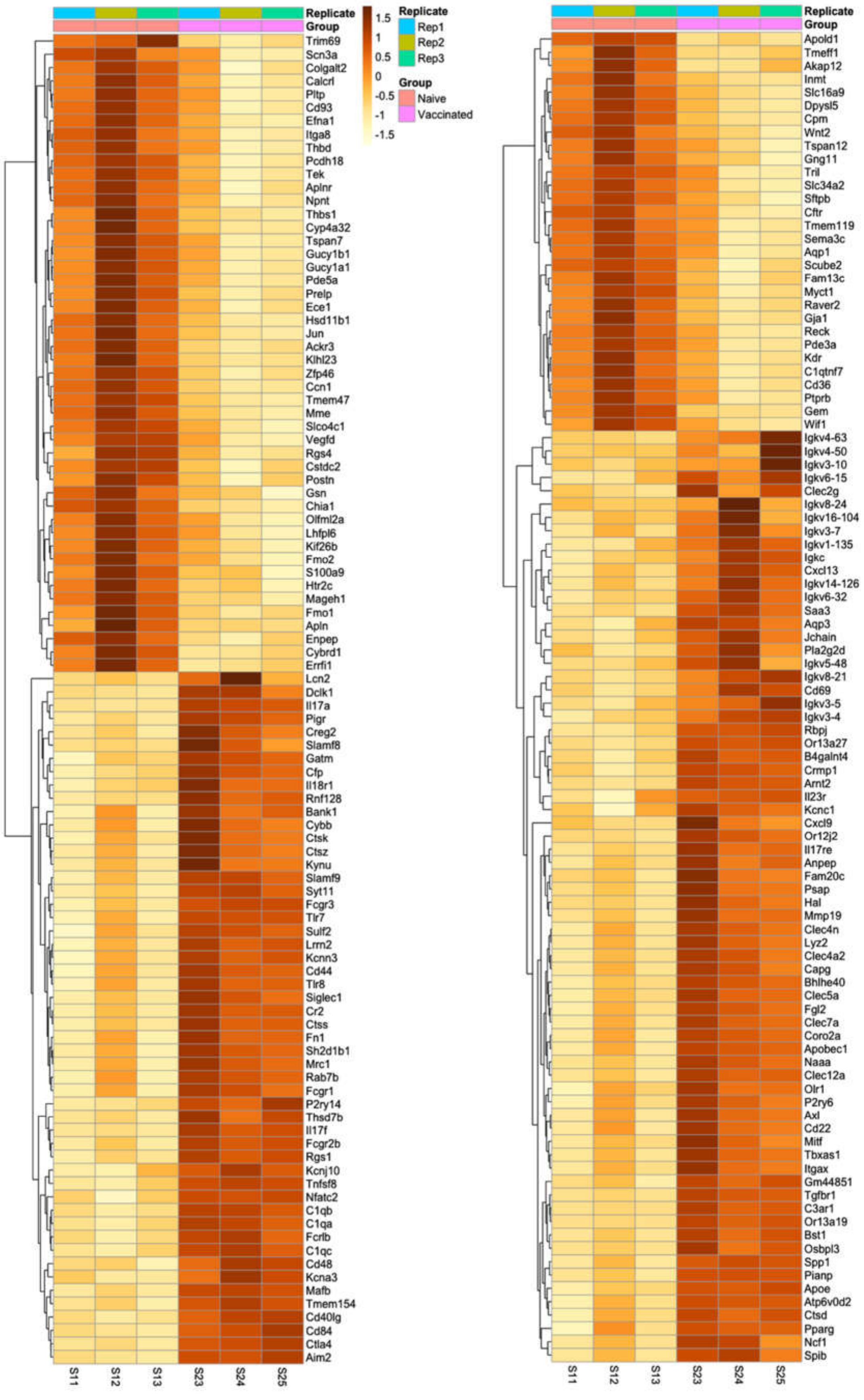

Replicate
Group
Trim69
Scn3a
Colgalt2
Calcrl
Pltp
Cd93
Efna1
Itga8
Thbd
Pcdh18
Tek
Aplnr
Npnt
Thbs1
Cyp4a32
Tspan7
Gucy1b1
Gucy1a1
Pde5a
Prelp
Ece1
Hsd11b1
Jun
Ackr3
Klhl23
Zfp46
Ccn1
Tmem47
Mme
Slco4c1
Vegfd
Rgs4
Cstdc2
Postn
Gsn
Chia1
Olfml2a
Lhfpl6
Kif26b
Fmo2
S100a9
Htr2c
Mageh1
Fmo1
Apln
Enpep
Cybrd1
Errfi1
Lcn2
Dclk1
Il17a
Pigr
Creg2
Slamf8
Gatm
Cfp
Il18r1
Rnf128
Bank1
Cybb
Ctsk
Ctsz
Kynu
Slamf9
Syt11
Fcgr3
Tlr7
Sulf2
Lrrn2
Kcnn3
Cd44
Tlr8
Siglec1
Cr2
Ctss
Fn1
Sh2d1b1
Mrc1
Rab7b
Fcgr1
P2ry14
Thsd7b
Il17f
Fcgr2b
Rgs1
Kcnj10
Tnfsf8
Nfatc2
C1qb
C1qa
Fcrlb
C1qc
Cd48
Kcna3
Mafb
Tmem154
Cd40lg
Cd84
Ctla4
Aim2
S11
S12
S13
S23
S24
S25
1.5
1
0.5
0
-0.5
-1
-1.5
Replicate
Rep1
Rep2
Rep3
Group
Naive
Vaccinated
Replicate
Group
Apold1
Tmeff1
Akap12
Inmt
Slc16a9
Dpysl5
Cpm
Wnt2
Tspan12
Gng11
Tril
Slc34a2
Sftpb
Cftr
Tmem119
Sema3c
Aqp1
Scube2
Fam13c
Myct1
Raver2
Gja1
Reck
Pde3a
Kdr
C1qtnf7
Cd36
Ptprb
Gem
Wif1
Igkv4-63
Igkv4-50
Igkv3-10
Igkv6-15
Clec2g
Igkv8-24
Igkv16-104
Igkv3-7
Igkv1-135
Igkc
Cxcl13
Igkv14-126
Igkv6-32
Saa3
Aqp3
Jchain
Pla2g2d
Igkv5-48
Igkv8-21
Cd69
Igkv3-5
Igkv3-4
Rbpj
Or13a27
B4galnt4
Crmp1
Arnt2
Il23r
Kcnc1
Cxcl9
Or12j2
Il17re
Anpep
Fam20c
Psap
Hal
Mmp19
Clec4n
Lyz2
Clec4a2
Capg
Bhlhe40
Clec5a
Fgl2
Clec7a
Coro2a
Apobec1
Naaa
Clec12a
Olr1
P2ry6
Axl
Cd22
Mitf
Tbxas1
Itgax
Gm44851
Tgfbr1
C3ar1
Or13a19
Bst1
Osbpl3
Spp1
Pianp
Apoe
Atp6v0d2
Ctsd
Pparg
Ncf1
Spib
S11
S12
S13
S23
S24
S25

Supplemental Figure S11. Heatmap of differentially expressed genes in young mice following vaccination. The expression levels of significantly upregulated and downregulated genes in young mice following vaccination were illustrated. Each row represents a single gene, and each column represents an individual sample. The color gradient from yellow to red indicates the range of gene expression levels, with yellow representing downregulated genes and red representing upregulated genes.
